# Motor and non-motor outcomes in patients with advanced Parkinson’s disease treated with levodopa/carbidopa intestinal gel: final results of the GREENFIELD observational study

**DOI:** 10.1007/s00415-019-09337-6

**Published:** 2019-05-27

**Authors:** Leonardo Lopiano, Nicola Modugno, Pietro Marano, Mariachiara Sensi, Giuseppe Meco, Paolo Solla, Graziano Gusmaroli, Filippo Tamma, Francesca Mancini, Rocco Quatrale, Roberta Zangaglia, Annarita Bentivoglio, Roberto Eleopra, Giuliana Gualberti, Gabriella Melzi, Angelo Antonini

**Affiliations:** 1 Department of Neuroscience, University of Torino, Torino, Italy; 2 IRCCS “Neuromed”, Pozzilli, Italy; 3 Casa Di Cura Madonna del Rosario, Raggruppamento Di Riabilitazione, Catania, Italy; 4 Neurology Unit, Azienda Ospedaliera Universitaria Sant’Anna, Cona, Ferrara, Italy; 5 Department of Neurology and Psychiatry (Parkinson’s Centre), Research Centre of Social Diseases (CIMS), Sapienza University, Rome, Italy; 6 Neurology Unit, Policlinico Universitario Monserrato, Cagliari, Italy; 7 Neurology Unit, Ospedale degli Infermi, Biella, Italy; 8 Neurology Unit, Miulli Hospital, Acquaviva delle Fonti, BA Italy; 9 UO Neurologia e Stroke-Unit e Laboratorio Neuroscienze, Istituto Auxologico San Luca, Milano, Italy; 10 Neurology Unit, Hospital Dell’Angelo, Mestre, VE Italy; 11 Parkinson’s Disease and Movement Disorders Unit, IRCCS Neurological National Institute C. Mondino, Pavia, Italy; 12 Institute of Neurology, Università Cattolica del Sacro Cuore, Rome, Italy; 13 Fondazione Policlinico Universitario A. Gemelli IRCCS, Rome, Italy; 14 Fondazione IRCCS Neurological Institute Carlo Besta, Milan, Italy; 15 AbbVie Srl, Campoverde, LT Italy; 16 Parkinson and Movement Disorders Unit, Department of Neuroscience, University of Padua, Padua, Italy

**Keywords:** Advanced Parkinson’s disease, Levodopa/carbidopa, Intestinal infusion, Motor symptoms, Quality of life, Routine patient care

## Abstract

**Introduction:**

The GREENFIELD observational study assessed the effect of levodopa/carbidopa intestinal gel (LCIG) on motor and non-motor symptoms, and the related impact on patient quality of life and caregiver burden up to 8 years.

**Methods:**

Final results of a large Italian cohort of patients who started LCIG in routine care between 2007 and 2014 are presented. Comparison between baseline (before LCIG) and follow-up visits on yearly basis (visit 2/3) is reported. Primary endpoint was Unified Parkinson’s Disease Rating Scale (UPDRS-IV) Item 39; secondary endpoints were UPDRS I and II, dyskinesia items, PD Quality of Life Questionnaire-39, Parkinson’s Disease Sleep Scale-2, Gait and Falls Questionnaire, Questionnaire on Impulsive Disorders, and Relative Stress Scale.

**Results:**

Overall, 145 patients from 14 centers were assessed with a mean time since LCIG start of 2.8 ± 1.7 years at visit 2. The mean UPDRS-IV item 39 score showed significant reductions compared to baseline (mean score 2.0 ± 0.81) at visit 2 (mean score 0.9 ± 0.69; − 55%; *p* < 0.001) and at visit 3 (mean score 1.0 ± 0.75; − 50%; *p* < 0.001). At visit 3, significant reductions were observed for dyskinesia duration score (− 28%; *p* < 0.001), dyskinesia disability (− 40%; *p* < 0.001), and painful dyskinesia (− 50%; *p* < 0.001). Overall, 40 (27.6%) patients experienced 49 serious adverse events which were considered related to PEG/J procedure or to device in 16.3% of the cases.

**Conclusions:**

The results of this study support the long-term efficacy of LCIG on PD symptoms as well as on activities of daily living. The adverse events were consistent with the established LCIG safety profile.

**Electronic supplementary material:**

The online version of this article (10.1007/s00415-019-09337-6) contains supplementary material, which is available to authorized users.

## Introduction

Parkinson’s disease (PD) is a chronic, progressive neurodegenerative disorder characterized by motor and non-motor symptoms that impair patient’s autonomy and quality of life (QoL), together with a consequent burden also on caregiver QoL [1].

Oral levodopa efficacy shortens as PD progresses; in fact, within 2–5 years up to 50% of patients already may experience some degree of motor complications, with 80–100% of PD patients developing motor complications after 10 years of dopaminergic therapy. This is mainly due to the progressive loss of striatal dopamine nerve terminals, the short levodopa half-life, the delayed gastric emptying and abnormal intestinal absorption [2–4].

Levodopa/carbidopa intestinal gel (LCIG) is continuously delivered via a percutaneous endoscopic gastrostomy with a jejunal extension (PEG-J) providing a more stable plasma concentration in patients with non-optimal control of motor fluctuations [5].

Initial evidence for the efficacy and tolerability of LCIG came from a number of small-sample studies.[6, 7]. Recently, three randomized clinical trials (RCTs) (one of them double blinded) have confirmed that LCIG reduced OFF time, increased ON time without increasing troublesome dyskinesias, and improved QoL [8–10]. The antidyskinetic effect of LCIG has been reported in the post hoc analyses of a 12-week double-blind study and of a 54-week open-label study on patients having at least 1 h per day of ON-time with troublesome dyskinesia [11]. Recently, a 6-month open-label pilot study, showing a 47% reduction of ON-time with troublesome dyskinesia, stated that LCIG has a substantial antidyskinetic effect and could be an alternative treatment also for PD patients with dyskinesia.[12].

Moreover, LCIG leads to significant improvements in motor and non-motor symptoms, in daily living activities and QoL also in patients with motor complications [13].

The long-term effectiveness of levodopa/carbidopa in APD patients on motor complications and QoL has been assessed in several routine clinical care studies up to 4 years of treatment duration [14–19]. Few studies have been conducted in large populations to assess the LCIG outcomes longer than 4 years [20, 21]. Therefore, in the current study, we recorded the clinical outcomes in a large cohort of APD patients receiving LCIG in routine clinical care to evaluate the effects of therapy on both motor and non-motor symptoms, and the related impact on patient QoL and caregiver burden from the initiation of LCIG therapy over a maximum exposure period up to 8 years. The interim analysis of this study comparing the changes in primary endpoint and UPDRS scores between visit 1 (V1) and baseline (BL) data was previously published [22]. Here, we present the final results of this study.

## Patients and methods

This post-marketing observational study was conducted in 14 Italian Movement Disorder Centers.

Treatment with LCIG was initiated in a routine patient care setting, according to the Summary of Product Characteristics, including the temporary naso-jejunal (NJ) phase to determine if the patient responded favorably to LCIG and to optimize the dose before treatment was initiated via PEG-J tube.

### Patient selection

Consecutive APD patients showing motor fluctuations despite the use of oral medication, who started LCIG infusion according to clinical practice between 2007 and 2014, were considered eligible for enrollment into the study.

Inclusion criteria were: to be already treated with LCIG, presence of adequate information about previous medical history and treatment, and presence of at least one fulfilled scale or questionnaire among those collected during the study. Patients could be enrolled at any time after LCIG treatment initiation. Exclusion criteria were the presence of any conditions that, at the physician’s discretion, could interfere with the long-term treatment with LCIG.

At the enrollment visit (V1), patient history and retrospective clinical data referred to previous conventional PD treatment, NJ phase and initiation of LCIG treatment via PEG-J were collected as BL data. During the same visit, current clinical parameters were also collected as V1 data. For the analysis, BL was defined as the last available data collected prior to NJ tube positioning.

### Study design and treatment

The study design included two patient populations: the retrospective population and the prospective population. The retrospective population included all patients who had been receiving treatment with LCIG for ≥ 1 year and up to 7 years before V1, with available BL retrospective assessment data for ≥ 1 year. The prospective population included all patients receiving treatment with LCIG for < 1 year before V1. Patients continuing with LCIG treatment for further 2 years after enrolment and with follow-up visits on yearly basis (visit 2, V2 and visit 3, V3 or at the last available visit) were included in the final analysis.

### Assessments

#### Effectiveness

The primary endpoint was the change from baseline to the last available follow-up (V2 or V3 or the last available visit) in the item-39 of the UPDRS-IV (proportion of waking day spent in OFF).

At V1 (enrolment visit), the following baseline data for secondary effectiveness measures were collected: demographics, medical history, previous PD treatments, NJ phase, LCIG dose of infusion at discharge from the hospital; the Hoehn and Yahr scale; the UPDRS I total score (in ON and OFF conditions), activities of daily living (ADL), as assessed by means of the UPDRS II total score (in ON and OFF conditions), UPDRS IV (total score), and items for dyskinesia duration (Item 32), dyskinesia severity (Item 33), painful dyskinesia (Item 34), and early-morning dystonia (Item 35). At the same visit, PD Quality of Life Questionnaire (PDQ-39), Parkinson’s Disease Sleep Scale (PDSS-2), Gait and Falls Questionnaire (GFQ), Questionnaire on Impulsive Disorders (QUIP-RS), and the Relative Stress Scale (RSS) for caregiver burden assessment were collected only for patients in treatment with LCIG for more than 1 year before the enrolment visit and for all patients with missing BL value.

The same assessments were repeated at V2 and at V3 including concurrent diseases and therapies, dosages and changes in LCIG daily infusion, and the economic and social impact of the familiar caregiver assistance. At each visit, the global efficacy on motor symptoms was rated by neurologist versus baseline by a three-point scale: improvement, no change, worsening. The patient’s judgment on LCIG benefit, was collected at each visit by means of a scale rating from 0 to 10 and grouped as follows: 0–2 very bad, 3–5 unsatisfactory, 6–8 satisfactory, 9–10 very good. Safety data were collected from enrolment visit onward.

#### Safety

Serious adverse events (SAE) were collected retrospectively at the enrolment visit (V1) and prospectively at each follow-up visit (V2 and V3). Adverse events (AE) were prospectively collected. Furthermore, all the Product Quality Complaints (PQC), with regard to the medical devices defined as communication that alleges deficiencies related to the identity, physical aspects, potency, expressed lack of effect, purity, packaging, durability, reliability, safety or performance, associated or not with an AE were retrospectively and prospectively collected.

### Statistical analysis

#### Sample size evaluation

Postulating a correlation between values at baseline and at last follow-up = 0 (conservative decision), a mean difference between baseline and last follow-up on the item 39 of UPDRS questionnaire of – 1.0, a SD of 2.0 and using a paired *t* test, 87 subjects were to be enrolled, with two-sided significance level = 5% and power = 90%. Considering a drop-out rate of 40% (according to the Italian clinical experience: 22% of drop-outs + 18% of deaths should be considered), at least 145 subjects were needed; therefore, a total of 150 patients were estimated to be enrolled.

#### Statistical analysis

All statistical analyses were carried out by means of the SAS® package (version 9.2). Continuous and categorical variables were summarized by descriptive statistics. Statistical significance was declared if the rounded *p* value was less than or equal to 0.05.

All patients from enrolled population that performed at least one post-baseline visit (V2 or V3 for prospective patients, V1 or V2 or V3 for retrospective patients) were included in the evaluable population. All analyses of efficacy variables were performed in the evaluable population.

For assessments over time (e.g., for all rating scales), the analysis by visit (V1–V3) was complemented by an analysis according to the “last-observation-carried-forward” (LOCF) principle: missing data at V3 have been replaced with the (non-missing) data recorded at V2. No baseline value (at V1) has been considered in the LOCF technique.

Comparisons between BL and each follow-up visit (with LOCF) of the efficacy endpoints were performed using a paired *t* test. Comparison between BL and each follow-up visit (with LOCF) of item 35 of UPDRS was performed by means of McNemar test. The same analysis was performed separately for the subgroups of prospective and retrospective patients. The comparison of the clinical indexes and the questionnaires scores during time was also confirmed through ANOVA for repeated measures on the population without LOCF.

To evaluate the impact of age of the patient and duration of disease on the UPDRS scores, a secondary analysis was performed by means of an analysis of covariance (ANCOVA) model with change from BL (with LOCF) as dependent variable, age of patient (dichotomous variable: ≤ 70 years; > 70 years), time since PD diagnosis (dichotomous variable: ≤ 13 years; > 13 years), and time since infusion ( < 1 year vs ≥ 1 year) as fixed effects and BL value as covariate. The difference between the adjusted means for age ≤ 70 years versus > 70 years and for time since PD diagnosis ≤ 13 years versus > 13 years were calculated with the associated 95% CI and *p* value.

## Results

**Fig. 1:**
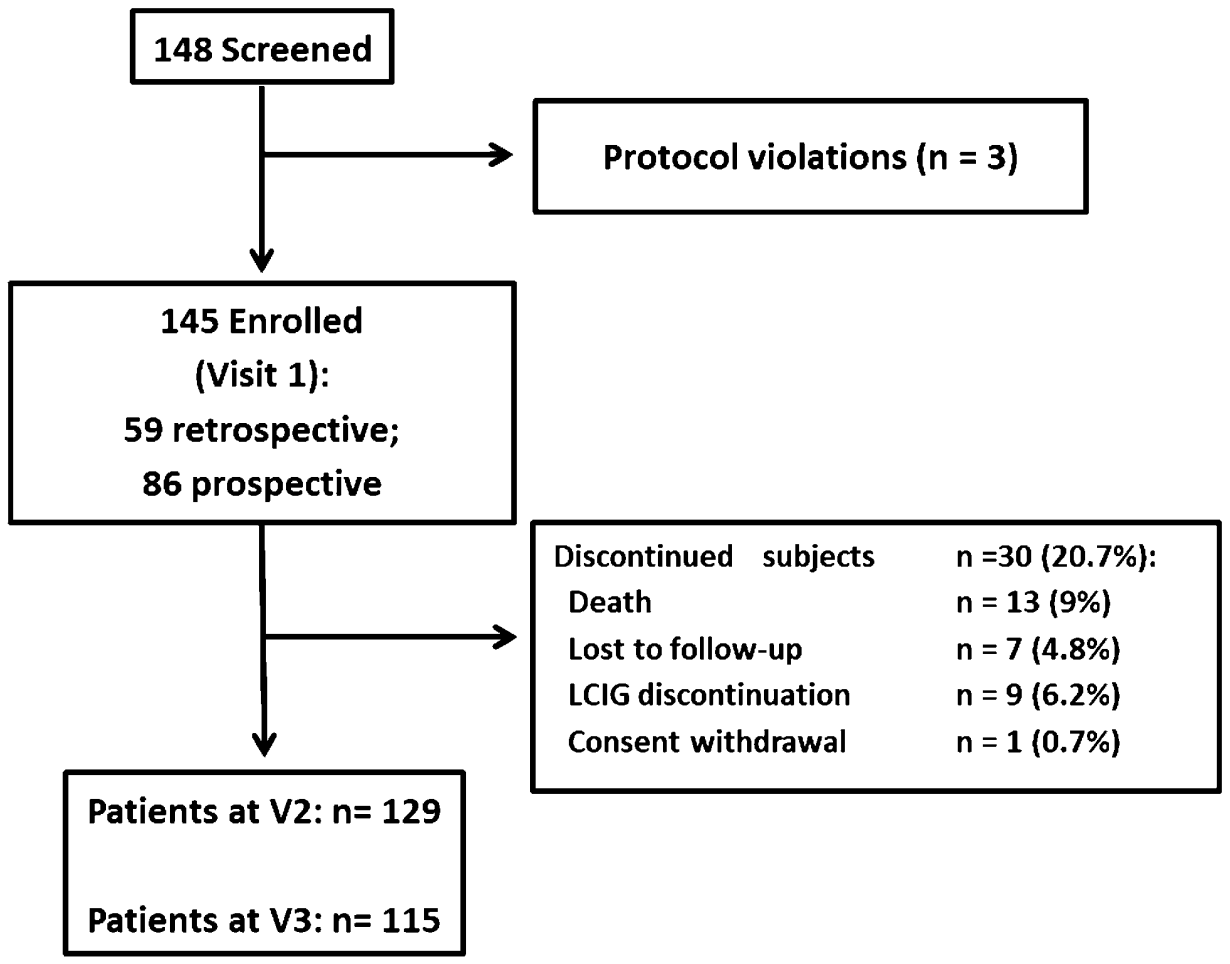
Patient disposition

From the start of enrollment in November 2012 through July 2014, a total of 148 patients were included among the participating centers. Three subjects were excluded, due to inclusion/exclusion criteria violation, so the enrolled sample consists of 145 patients and 137 were evaluable. Across the study, 30 patients discontinued for different reasons and 115 patients completed the 24-month follow-up (Fig. 1). Considering that no differences in the primary and secondary efficacy measures were found between the retrospective (*N* = 59) and prospective populations (*N* = 86; 78 evaluable), we present here the results of the total population. In the total evaluable population, BL values were the data recorded before naso-jejunal phase or, if missing, data recorded at V1.

Demographic, medical history, occupational status, and previous advanced PD medications before the initiation of LCIG are summarized in Table 1. The mean age of patients ( ± SD) was 70.4 ± 7.7 years, and the mean PD duration and the mean time since the onset of motor fluctuations were, respectively, 14.6 ± 6.5 and 5.9 ± 3.9 years.

**Table 1:** Demographic and clinical characteristics of the study population

Parameters	Value	Range
*Demographics*	*n* = 145	
Gender		
Female, *n* (%)	72 (49.7%)	
Male, *n* (%)	73 (50.3%)	
Age (years)	70.4 ± 7.7	49–90
Age		
< 65 years, *n* (%)	30 (20.7%)	
≥ 65 years, *n* (%)	115 (79.3%)	
Age > 70 years, *n* (%)	78 (53.8%)	
*PD medical history*		
Age at PD diagnosis (years)	55.7 ± 9.27	
PD duration (years)	14.61 ± 6.58	1.3–46.7
PD duration ≤ 13 years	72 (49.7%)	
PD duration > 13 years	73 (50.3%)	
Time since onset of motor fluctuations (years) (n = 143) (mean ± SD)	5.9 ± 4.0	1–21
Previous use of invasive treatments (before LCIG infusion)	*N*(%)	Daily dose, mean ± SD
Previous deep brain stimulation	3 (2.1%)	NA
Apomorphine SC (pump) (mg)	14 (9.7%)	86.29 (46.38)
Apomorphine stylo (mg)	7 (4.8%)	6.5 (10.6)

The antiparkinsonian medications with the daily dosages prior to start LCIG infusion are reported in Table 2; oral levodopa was the most commonly used antiparkinsonian medication (96.6% of patients), at a mean daily dose of 818.17 ± 404.4 mg.

**Table 2:** Use of antiparkinsonian medications before and during LCIG at each visit

Antiparkinsonian medications	Before LCIG start (*N* = 145)		Visit 1 (*N *= 145)		Visit 2 (*N* = 129)		*Visit 3 (N* = *115)*	
	*N* (%)	Daily dose (mg) mean ± SD	*N* (%)	Daily dose(mg) mean ± SD	*N* (%)	Daily dose (mg), mean ± SD	*N* (%)	Daily dose (mg) mean ± SD
Oral levodopa	140 (96.6%)	818.74 ± 404.4	5 (3%)—during the day 34 (23%)—at night	150.0 ± 70.7 during the day 160.3 ± 72.5 at night	4 (3%)—during the day 30 (23%)—at night	475.0 ± 518.8 during the day 164.2 ± 56.3 at night	4 (3%)—during the day 30 (26%)—at night	137.5 ± 75.0 during the day 186.7 ± 73.0 at night
Dopamine agonists	93 (64.1%)	6.38 ± 5.6	42 (29%)	5.6 ± 3.8	39 (30%)	5.1 ± 3.3	28 (24%)	6.6 ± 5.6
COMT inhibitors	64 (44.1%)	590.7 ± 337.4	16 (11%)	253.1 ± 105.6	16 (12%)	215.6 ± 67.6	14 (12%)	196.4 ± 69.2
MAO inhibitors	21 (14.5%)	2.33 ± 3.31	5 (3%)	3.6 ± 3.9	5 (4%)	5.8 ± 5.07	5 (4%)	3.6 ± 3.9
Amantadine	25 (17.2%)	190.6 ± 112.6	8 (6%)	237.5 ± 91.6	12 (9%)	200.0 ± 95.3	10 (9%)	220.0 ± 113.5

Data presented in mean ± standard deviation (SD) or number (%)

*LCIG *levodopa/carbidopa intestinal gel, *PD* Parkinson’s disease, *SC* subcutaneous

The primary reasons to switch from oral antiparkinsonian therapy to LCIG were disabling OFF periods in 115 patients (79%) and uncontrolled dyskinesia in 32 patients (25%). At the last visit, the mean time since the LCIG start was 2.8 ± 1.66 years with a maximum treatment period of 8 years. The mean continuous infusion rate at the discharge from hospital was 3.21 ± 1.09 ml/h and it remained stable at the following visits (respectively, 3.23 ± 1.03 and 3.31 ± 1.28 at V2 and V3). LCIG was infused for 16 h per day, while only one patient at V1 and three patients at V3 reported a 24-h infusion.

### Efficacy

**Fig. 2:**
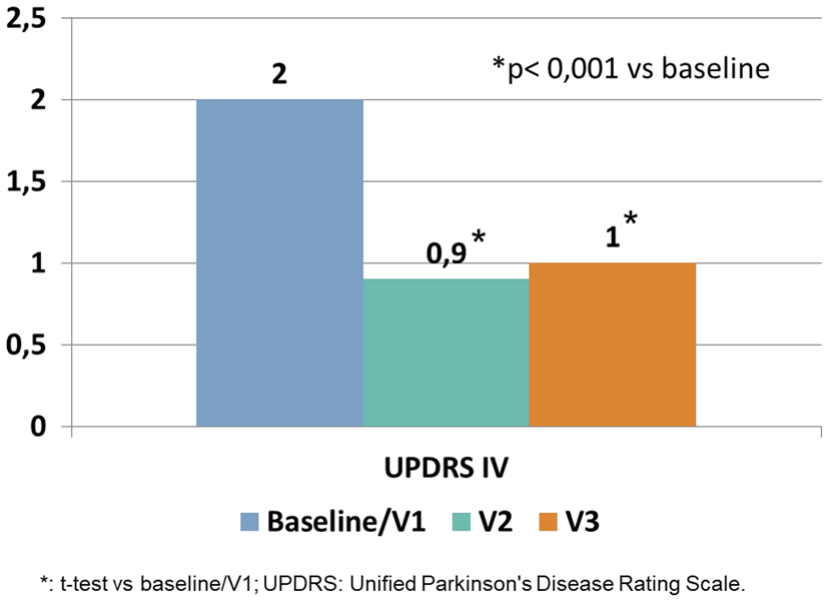
UPDRS-Part IV mean subscores at baseline/V1 (*N* = 139), V2 (*N* = 129) and V3 (*N* = 115)

The mean UPDRS-IV item 39 score showed significant and sustained reductions at V2 compared to BL (mean BL score 2.0 ± 0.81, mean V2 score 0.9 ± 0.69; − 55%; *p* < 0.001) and also compared to V3 (mean score 1.0 ± 0.75; − 50%; *p* < 0.001), as reported in Fig. 2.

**Fig. 3:**
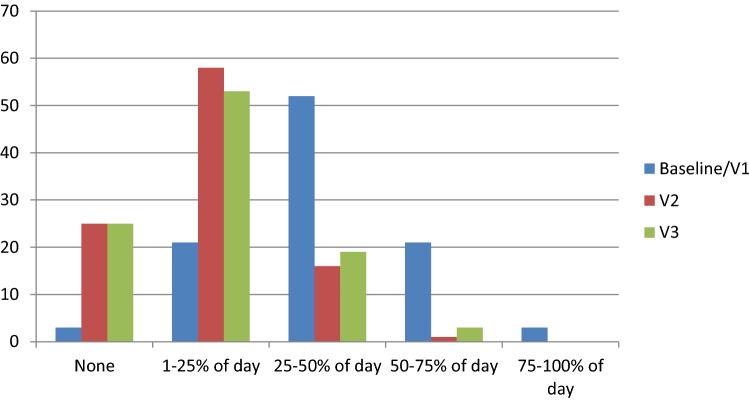
Distribution of UPDRS-IV Item 39 at Visit 2 (V2) and Visit 3 (V3) compared to baseline or V1

Furthermore, at each visit, respectively, 83% and 78% of the patients showed less than 25% of daily time spent in OFF while at BL the majority of them (76%) had more than 25% of daily time in OFF (Fig. 3).

The mean LCIG treatment period at the primary endpoint assessment (V3) was 3.1 ± 1.5 years in the general population, while in the retrospective population the mean LCIG treatment period was 3.7 ± 1.7 years at V2 and 4.5 ± 1.5 years at V3.

At the last follow-up visit (V3), significant reductions compared to BL were observed for dyskinesia duration score (− 28% UPDRS IV Item 32; *p* < 0.001), dyskinesia disability (− 40% UPDRS IV Item 33; *p* < 0.001), and painful dyskinesia (− 50% UPDRS IV Item 34; *p* < 0.001) with a similar trend at each follow-up visit. The mean score reductions for each of the above-mentioned parameters and the corresponding *p* values are reported in Table 3, along with the ANOVA results for repeated measures.

**Table 3:** Complications of therapy (UPDRS IV), UPDRS-I, ADL and H&Y stage at baseline (before LCIG treatment) and at each follow-up visit, without LOCF

	BL mean score ( ± SD) (Range)	N	Visit 2	N	Visit 3	N	Reduction at V3 vs BL	*p value at V2 vs BL **p value at V3 vs BL	*p* ANOVA for repeated measures
UPDRS IV total score (Items 32–42)	8.2 (3.27) (0–18)	134	4.7 (2.88) (0–14)	124	4.9 (3.12) (0–14)	112	− 40%	* < 0.001 ** < 0.001	< 0.001
Dyskinesia duration (Item 32)	1.8 (1.04) (0–4)	134	1.2 (0.89) (0–4)	127	1.3 (1.02) (0–4)	113	− 28%	* < 0.001 ** < 0.001	< 0.001
Dyskinesia disability (Item 33)	1.5 (1.11) (0–4)	133	0.9 (1.02) (0–4)	124	0.9 (1.01) (0–4)	112	− 40%	* < 0.001 ** < 0.001	< 0.001
Painful dyskinesia (Item 34)	0.8 (0.97) (0–4)	133	0.4 (0.69) (0–4)	124	0.4 (0.73) (0–4)	112	− 50%	* < 0.001 ** < 0.001	< 0.001
Presence of early morning dystonia (Item 35)	58 (44%) (0–1)	133	35 (28%)	127	28 (25%)	113	− 43%	*0.002 ** < 0.001	< 0.001 (Logit)
OFF time duration (Item 39)	2.0 (0.81) (0–4)	137	0.9 (0.69) (0–3)	127	1.0 (0.75) (0–3)	114	− 50%	* < 0.001 ** < 0.001	< 0.001
UPDRS I total score									
OFF	6.8 (4.75) (0–16)	79	5.9 (3.86) (0–16)	81	6.0 (3.71) (1–16)	65	− 12%	*0.054	0.006
ON	4.3 (3.08) (0–12)	96	3.6 (2.71) (0–12)	126	3.8 (2.78) (0–12)	110	− 12%	**0.055	0.008
UPDRS II (ADL) total score									
OFF	29.2 (9.63) (0–48)	97	25.7 (8.49) (8–43)	86	25.5 (8.76) (8–46)	72	− 13%	* < 0.001 **0.003	< 0.001
ON	18.2 (9.39) (0–39)	111	16.2 (8.49) (1–43)	127	16.2 (8.45) (1–34)	109	− 11%	*0.003 **0.078	< 0.001
UPDRS V (Hoehn and Yahr)									
OFF	3.99 (0.82) (2–5)	120	3.59 (0.85) (1–5)	100	3.7 (0.81) (2–5)	88	− 7%	*< 0.001 **0.003	< 0.001
ON	3.07 (0.75) (1–5)	135	2.72 (0.77) (0–5)	128	2.77 (0.83) (0–5)	115	− 10%	* < 0.001 ** < 0.001	< 0.001

*ADL* activities of daily living, *BL* baseline, *LCIG* levodopa/carbidopa intestinal gel, *SD* standard deviation; UPDRS, United Parkinson’s Disease Rating Scale

The UPDRS-IV (Part A + B) was significantly reduced compared to BL (mean baseline score 8.2 ± 3.27) both at V2 (mean score 4.7 ± 2.88; *p* < 0.001) and at V3 (mean score 4.9 ± 3.12; *p* < 0.001).

Twenty-five percent of the patients reported early morning dystonia (UPDRS IV Item 35) at V3 while this percentage was higher at BL (44% of the patients, *p* < 0.001).

There was a significant improvement through the whole study duration, on UPDRS II in OFF state that was reduced from 29.2 ± 9.63 at BL to 25.7 ± 8.49 (*p* < 0.001) at V2 and to 25.5 ± 8.76 at V3 (*p* = 0.003) (Table 3).

### Quality-of-life and PD-associated symptoms

The results on QoL and PD-associated symptoms questionnaires at each visit showed a different behavior in the retrospective and prospective population. The prospective population performed the assessments both at BL (close to LCIG start) and at each follow-up visit, while most of the assessments were not performed close to LCIG start for the retrospective population. Therefore, we show in Table 4 the results of the prospective population and in Supplementary Table 1 those of the retrospective population.

**Table 4:** Outcome of non-motor symptoms and PD-associated symptoms at baseline and after LCIG at each treatment visit

Prospective population without LOCF	Baseline/V1 Mean ± SD	Visit 2 Mean ± SD	Visit 3 Mean ± SD	*p* vs baseline	*p* ANOVA for repeated measures
**PDQ-39 (score 0–156)**	72.3 ± 23.8	64.7 ± 25.4*	67.3 ± 26.4**	*p < 0.001 **p < 0.05	< .0.001
**PDSS-2 (score 0–60)**	25 ± 10.4	22.5 ± 9.9*	22.7 ± 10.1	*p < 0.01	0.016
**GFQ (score 0–64)**	29.7 ± 13.3	26.5 ± 13.1*	26.1 ± 12	*p < 0.05	0.079
**QUIP-RS (score 0–112)**	10.4 ± 16.6	7.8 ± 13*	7.1 ± 10.1*	*p < 0.05	0.011
QUIP-gambling (score 0–16)	1.2 ± 3.3	0.9 ± 2.6	0.5 ± 1.7	NS	0.085
QUIP-sexual behavior	1.5 ± 2.8	1.1 ± 2.5*	1.2 ± 2.7	*p < 0.05	0.140
QUIP-buying	1.5 ± 3.2	1.1 ± 2.3	0.8 ± 1.8	NS	0.100
QUIP-eating	2.2 ± 4	1.8 ± 3.6	1.5 ± 3*	*p < 0.05	0.051
QUIP-hobbism	1.3 ± 2.7	1.2 ± 26	1.3 ± 2.5	NS	0.815
QUIP-punding	1.3 ± 3.4	1 ± 2.6	0.7 ± 1.9	NS	0.128
QUIP-Medication use	1.4 ± 3.5	0.7 ± 2.1*	1.2 ± 3	*p < 0.01	0.093
**RSS-2 (score 15–75)**	40.2 ± 12.4	39 ± 13.3	38.3 ± 13	NS	0.237
RSS/personal distress	16.5 ± 5.5	16.1 ± 5.7	15.9 ± 5.6	NS	0.467
RSS/negative feeling	9.2 ± 3.6	8.9 ± 3.7	8.7 ± 3.5	NS	0.085
RSS/life upset	14.5 ± 5	14 ± 5.1	13.7 ± 5	NS	0.455

ADL, activities of daily living; BL, baseline; LCIG; levodopa/carbidopa intestinal gel; SD, standard deviation; UPDRS, United Parkinson’s Disease Rating Scale

*p value at V2 vs BL, **p value at V3 vs BL

The PDQ-39 showed significant improvements at V2 (mean score 64.7 ± 25.4; *p* < 0.001) and at V3 (mean score 67.3 ± 26.4; *p* < 0.05) compared to BL (mean score 72.3 ± 23.8) (Table 4).

Significant improvement in PDSS-2, GFQ and QUIP-RS was also observed across all study visits. Among the QUIP-RS sub-items, sexual behavior, eating, and medication use showed a significant improvement compared to baseline only in the prospective population. The RSS for caregiver burden assessment did not show significant changes during the study compared to BL. The corresponding ANOVA analysis for repeated measurement confirmed these results except for GFQ and QUIP-Medication use (Table 4).

**Fig. 4:**
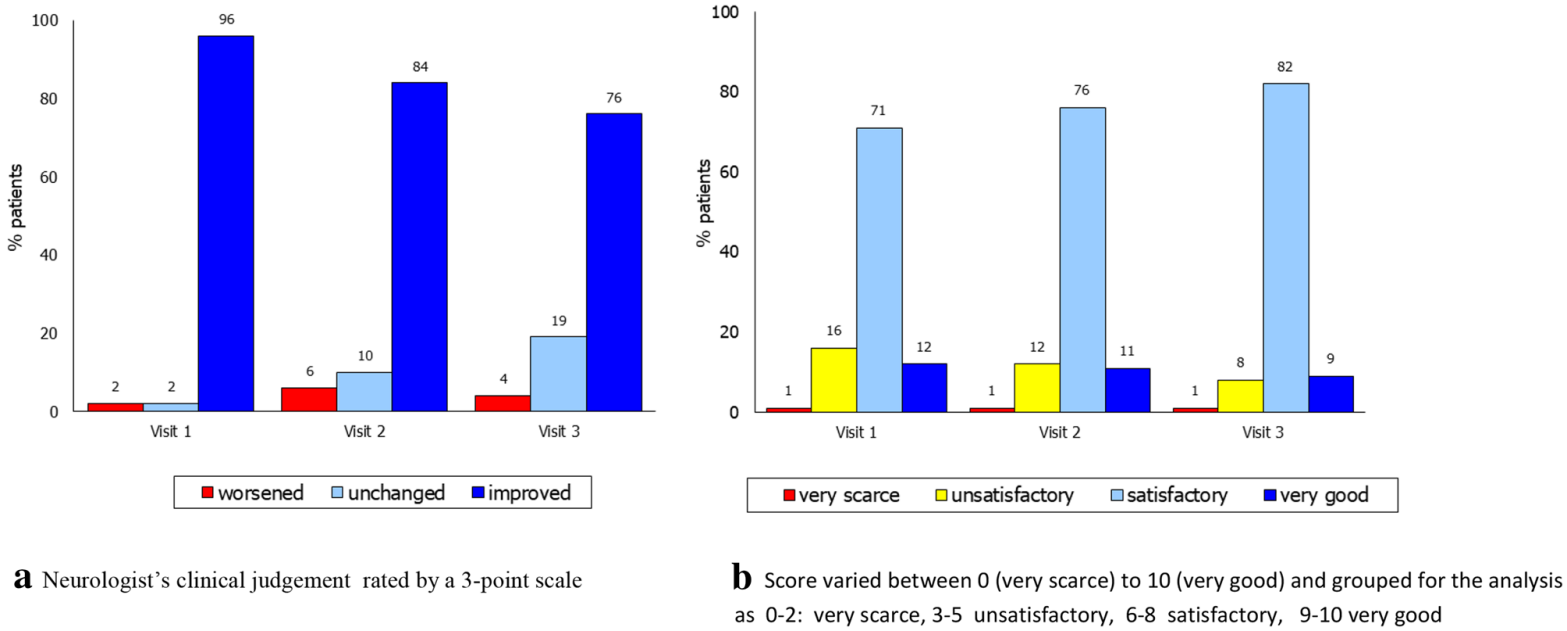
Global efficacy of LCIG made by neurologists vs baseline (**a**) and patient ‘s judgement on LCIG therapy at each visit (**b**). **a** Neurologist’s clinical judgement rated by a three-point scale. **b** Score varied between 0 (very scarce) and 10 (very good) and grouped for the analysis as 0–2 very scarce, 3–5 unsatisfactory, 6–8 satisfactory, 9–10 very good

Moreover, the clinicians considered 96% of the patients as “improved” since the first visit while only in 4% of the cases they reported a clinical worsening. Similarly, the patients’ judgment on their QoL was “very good” or “satisfactory” in 91% of the cases at the last visit with a positive trend across the study (Fig. 4).

### Secondary efficacy analysis

The sub-analyses according to age, disease duration, and time since infusion (< 1 year vs ≥ 1 year) showed that, compared to BL, there was a significant improvement at V2 and V3 of the UPDRS II score during “ON” (respectively, *p* = 0.001 and *p* = 0.006) and at V3 of the UPDRS IV total score (*p* = 0.02) in patients with shorter ( ≤ 13 years) compared with those with longer PD duration ( > 13 years). A significant improvement was observed at V2 of the UPDRS-I in OFF (*p* = 0.043) in patients aged ≤ 70 years; on the contrary, the same patients showed a significant worsening at V3 in UPDRS-IV total score (*p* = 0.024), UPDRS-IV item 32 (*p* = 0.02), and UPDRS-IV item 33 (*p* = 0.011) compared with those being more than 70 years old, as reported in Table 5. Moreover, comparison based on the time since infusion, both at V2 and at V3, did not show differences in the majority of the UPDRS items except for a significant improvement in UPDRS-I ON (*p* = 0.036 and *p* = 0.004) and in UPDRS-II ON (*p* = 0.012 and *p* = 0.019), and in UPDRS-IV item 33 (*p* = 0.012 at V2) in patients with less than 1 year of LCIG infusion (Table 5).

**Table 5:** Impact of age of the patient, duration of disease and of time since infusion on the UPDRS scores ANCOVA model

All population—ANCOVA model	Visit 2—year 1			Visit 3—year 2		
	Mean change	95% confidence Interval	*p* value	Mean change	95% confidence interval	*p* value
UPDRS item 39						
Age ( < = 70 vs > 70 years)	0.01	− 0.27 to 0.265	0.947	0.16	− 0.11 to 0.43	0.242
Time since PD diagnosis ( < = 13 vs > 13 years)	− 0.15	− 0.41 to 0.10	0.245	− 0.20	− 0.47 to 0.07	0.142
Baseline			0.798			0.153
Time since infusion (prospective vs retrospective)	0.05	− 0.21 to 0.31	0.694	0.14	− 0.13 to 0.40	0.302
UPDRS I ‘ON’						
Age ( < = 70 vs > 70 years)	0.37	− 0.64 to 1.38	0.473	0.32	− 0.82 to 1.456	0.576
Time since PD diagnosis ( < = 13 vs > 13 years)	− 0.73	− 1.72 to 0.27	0.151	− 0.27	− 1.39 to 0.85	0.633
Baseline			0.001			0.046
**Time since infusion (prospective vs retrospective)**	− **1.12**	− **2.16** to − **0.07**	**0.036**	− **1.72**	− **2.88** to − **0.56**	**0.004**
UPDRS I ’OFF’						
**Age ( < = 70 vs > 70** **years)**	− **1.47**	− **2.89** to − **0.05**	**0.043**	− 1.24	− 3.29 to 0.81	0.231
Time since PD diagnosis ( < = 13 vs > 13 years)	0.91	− 0.42 to 2.4125	0.177	− 0.04	− 1.91 to 1.84	0.967
Baseline			< 0.001			< 0.001
Time since infusion (prospective vs retrospective)	− 1.16	− 2.47 to 0.15	0.081	− 1.20	− 3.01 to 0.60	0.187
UPDRS II ’ON’						
Age ( < = 70 vs > 70 years)	1.10	− 1.59 to 3.78	0.420	1.23	− 1.65 to 4.11	0.399
**Time since PD diagnosis ( < = 13 vs > 13** **years)**	− **4.59**	− **7.30** to − **1.88**	**0.001**	− **4.09**	− **6.99** to − **1.19**	**0.006**
Baseline			< 0.001			< 0.001
**Time since infusion (prospective vs retrospective)**	− **3.52**	− **6.25** to − **0.78**	**0.012**	− **3.53**	− **6.46** to − **0.59**	**0.019**
UPDRS II ‘OFF’						
Age ( < = 70 vs > 70 years)	− 2.95	− 6.29 to 0.39	0.083	− 1.53	− 5.11 to 2.04	0.396
Time since PD diagnosis ( < = 13 vs > 13 years)	− 2.25	− 5.52 to 1.02	0.175	− 3.08	− 6.59 to 0.42	0.084
Baseline			< 0.001			< 0.001
Time since infusion (prospective vs retrospective)	− 2.44	− 5.79 to 0.92	0.152	− 1.95	− 5.54 to 1.65	0.284
UPDRS IV (A + B)						
**Age ( < = 70 vs > 70** **years)**	0.32	− 0.72 to 1.36	0.545	**1.18**	**0.16** to **2.21**	**0.024**
**Time since PD diagnosis ( < = 13 vs > 13** **years)**	− 0.89	− 1.93 to 0.15	0.094	− **1.22**	− **2.24** to − **0.19**	**0.020**
Baseline			0.005			< 0.001
Time since infusion (prospective vs retrospective)	− 0.63	− 1.68 to 0.42	0.236	− 0.60	− 1.62 to 0.43	0.254
UPDRS Dyskinesia score: ITEM 32						
**Age ( < = 70 vs > 70** **years)**	0.19	− 0.13 to 0.50	0.246	**0.41**	**0.06** to **0.76**	**0.020**
Time since PD diagnosis ( < = 13 vs > 13 years)	− 0.13	− 0.45 to 0.18	0.591	− 0.23	− 0.58 to 0.11	0.186
Baseline			0.001			0.015
Time since infusion (prospective vs retrospective)	− 0.09	− 0.41 to 0.23	0.591	0.02	− 0.32 to 0.37	0.895
UPDRS Dyskinesia score: ITEM 33						
**Age ( < = 70 vs > 70** **years)**	0.12	− 0.25 to 0.49	0.527	**0.45**	**0.11** to **0.80**	**0.011**
Time since PD diagnosis ( < = 13 vs > 13 years)	− 0.11	− 0.49 to 0.26	0.544	− 0.14	− 0.48 to 0.21	0.443
Baseline			0.050			0.081
**Time since infusion (prospective vs retrospective)**	− **0.48**	− **0.85 to** − **0.11**	**0.012**	− **0.4**	− **0.69** to **0.01**	**0.055**
UPDRS Dyskinesia score: ITEM 34						
Age ( < = 70 vs > 70 years)	0.021	− 0.22 to 0.25	0.899	0.06	− 0.19 to 0.30	0.658
Time since PD diagnosis ( < = 13 vs > 13 years)	0.06	− 0.18 to 0.29	0.636	− 0.13	− 0.38 to 0.12	0.300
Baseline			< 0.001			< 0.001
Time since infusion (prospective vs retrospective)	− 0.14	− 0.38 to 0.09	0.232	0.02	− 0.23 to 0.27	0.873

Variables in bold are statistical significant for at least *p* < 0.05

### Adverse events

Overall, 40 (27.6%) patients experienced one or more SAEs (Table 6). The most frequently reported SAE, among those reported with a frequency higher than 1%, during LCIG infusion period were pneumonia (2.8%), femur fracture and cardiac failure (2.1%), peripheral neuropathy (1.4%), worsening of PD, fasciitis, and peritonitis (1.4% each). Only 8 out of 49 SAEs (16.3%) were related to PEG/J procedure or to device. (Table 6). In 12 out of 145 patients (8.3%) the AEs lead to discontinuation, as reported in Table 6. Fifty-four patients (37.2%) experienced PQCs; the most frequent were tube occlusion in 11 cases, dislocation of duodenal tube in 7 cases and phytobezoar in 5 cases (Table 6).

**Table 6:** Overall safety in the retrospective and prospective data collection cohorts

N. of patients with SAEs	40 (27.6%)
Event by preferred term	N. Events
Pneumonia	4 (2.8%)
Femur fracture	3 (2.1%)
Cardiac failure	3 (2.1%)
Cardiac arrest	2 (1.4%)
Peripheral neuropathy	2 (1.4%)
Worsening of PD	2 (1.4%)
Peritonitis	2 (1.4%)
Death	2 (1.4%)
Fasciitis	2 (1.4%)
Hip fracture	1 (0.7%)
Humerus fracture	1 (0.7%)
Cerebral haematoma	1 (0.7%)
Headache	1 (0.7%)
Hyperkinesia	1 (0.7%)
Acute myocardial infarction	1 (0.7%)
Gastric ulcer	1 (0.7%)
Haematemesis	1 (0.7%)
Inguinal hernia	1 (0.7%)
Intestinal obstruction	1 (0.7%)
Agitation	1 (0.7%)
Visual hallucination	1 (0.7%)
Suicide attempt	1 (0.7%)
Abnormal weight loss	1 (0.7%)
Hypoglycemia	1 (0.7%)
Acute pulmonary oedema	1 (0.7%)
Pulmonary embolism	1 (0.7%)
Anemia	1 (0.7%)
Deep vein thrombosis	1 (0.7%)
Related to PEG/J procedure or to device	
Wrong technique in drug usage process	2 (1.4%)
Gastrostomy tube site complication	1 (0.7%)
Pyrexia	1 (0.7%)
Administration site infection	1 (0.7%)
Stoma site infection	1 (0.7%)
Device occlusion	1 (0.7%)
Medical device complication	1 (0.7%)
Total SAEs	49
AEs leading to discontinuation	N. patients
Any AE leading to discontinuation	12 (8.3%)
Device occlusion/device complication	2 (1.4%)
Abnormal weight loss/hypoglycemia	2 (1.4%)
Fasciitis	2 (1.4%)
Peripheral sensory neuropathy	2 (1.4%)
Cardiac arrest	1 (0.7%)
Peritonitis	1 (0.7%)
Hallucination, visual	1 (0.7%)
Acute pulmonary oedema	1 (0.7%)
Product quality complaints during the study	
PQC experienced	54 (37.2%)
Event by preferred term	N. Events
Complaints associated with an adverse events	43 (29.7%)
Complaints associated with an ADR	29 (20%)
Complaints associated with a SAE	14 (9.7%)
Risks of PEG insertion	4 (2.8%)
Immediate peristomal infections	2
Bleedings and injury of internal organs	1
Infections including peritonitis and pneumoperitoneum	1
Device Complications (Infusion system)	30 (20.7%)
Accidental removal of PEG tube	2
Tube occlusion	11
Dislocation of duodenal tube	7
Tube rupture, accidental removal, device leakage	5
Phytobezoar	5
Surgical procedure	13 (9%)
Encrusted dressing	1
Signs of inflammation	3
Granulation tissue	9
Pump complication/rupture	8 (5.5%)

Thirteen deaths (9%) occurred during the observational period, mainly for cardiac reasons (six cases). For 12 deaths, a reasonably possible relationship has been reported.

## Discussion

Here, we report the final results from the largest cohort of patients with APD treated with LCIG in routine clinical practice from 14 Italian Movement Disorder Centers. The APD patients in this study were affected by motor fluctuations and dyskinesia not optimally controlled by standard oral or transdermal therapies. The previously published interim analysis of this study showed a significant reduction in total daily OFF time after a mean of 1.4 years of LCIG therapy with a magnitude of improvement consistent with the results reported in previous studies [22].

Results from this final analysis of up to 8 years of LCIG treatment (with a mean duration of 3 years) showed significant improvements on motor fluctuations, ADL and QoL. The magnitude of OFF reduction observed in our study is in agreement with the recently published data, where OFF reductions varied from 48 to 67% [8, 12, 20, 21, 23–25].

Moreover, the high percentage of patients reporting a UPDRS-IV item-39 score between 0 and 1 during LCIG infusion (approximately 80% at each visit) is also in line with the recently reported data from the PREDICT cross-sectional study where the percentage for this score was 81% for patients treated with LCIG and only 17% in patients treated with oral standard PD therapies [26]. The clinical relevance of this finding is further supported by the significant improvement of all UPDRS IV items related to dyskinesia and the total score of UPDRS IV.

The results of previous clinical studies on LCIG infusion have already indicated that this is an effective therapeutic strategy for improvements of motor symptoms (reduction in OFF time, increase in ON time without disabling dyskinesia, reduction of troublesome dyskinesia) [17, 27], non-motor symptoms (somnolence, fatigue, cardiovascular and urinary function, tremor at rest), and QoL [10, 28–30].

We observed significant improvements in UPDRS II for ADL, comparable to those reported in a 12-month prospective observational study including APD patients treated with LCIG as they start presenting with motor complications, having either 2–4 h of OFF time or 2 h of dyskinesia daily. In this study, patients experienced significant improvements from baseline in non-motor symptoms, with corresponding improvements in ADL up to 12 months [13].

Significant improvements compared to baseline were observed in the prospective population for QoL assessed by PDQ-39, quality of sleep assessed by PDSS-2, gait and balance disorders assessed by GFQ, and compulsive behavior by QUIP-RS with a significant impact on sexual behavior, eating, and medication use habit. A significant efficacy on these non-motor complications was previously also reported by Fasano et al. in a small group of patients treated with LCIG for 24 months [31]. Our results on sleep are in line with previously published data showing that LCIG improved the quality of sleep and produced a less fragmented sleep pattern measured by polysomnography after 6 months of therapy [32, 33].

It is interesting to note that in addition to the already established efficacy on motor and non-motor symptoms, there are clinically meaningful data regarding the positive effect of LCIG on axial symptoms. In fact, it has recently been reported in a retrospective study on 32 advanced PD patients, that LCIG has a favorable effect on freezing of gait (FoG), mainly represented by Pseudo-ON FoG and OFF-type-FoG [25, 34]. Moreover, LCIG was shown to be effective in seven PD patients with prominent episodes of freezing refractory to oral therapy [35].

In the sub-analyses of this study, we observed that patients with a shorter PD duration ( ≤ 13 years) had a better outcome on motor complications and ADL compared to patients with a longer PD duration, while the age is discriminant only for motor complications which are slightly more disabling in younger compared to older patients.

The finding that patients with shorter disease duration had a greater efficacy in ADL is in line with the results obtained in the MONOTREAT study, where ADL score was significantly improved starting from 3 months of treatment in patients with 13 years of PD duration and with short daily OFF time period (2–4 h) or dyskinesia period (2 h of troublesome or non-troublesome dyskinesia) [13].

These evidences strongly suggest that, in advanced PD patients, early treatment with LCIG should be carefully and promptly evaluated to improve motor complications and daily living activities.

The safety results were consistent with the established profile of LCIG [30, 21, 36, 37]. The percentage of SAEs was in the range of the adverse drug reactions reported in the complete GLORIA observational registry [24], with device and procedure-related events more frequently reported. These observations must be taken into consideration on the necessity for close monitoring in the immediate post PEG/J positioning period and in the long-term follow-up.

This is the first Italian study with data from a large population followed for a long period of time. Since this investigation was observational, with the collection of data recorded during routine medical care, we consider these outcomes to be close to “real-world’ clinical practice and consistent with results generated in controlled short-term clinical studies. The mean treatment period in this cohort was approximately 3 years; clinical outcomes were followed through 24 months of follow-up in this cohort of 145 patients with APD to assess the benefits of LCIG infusion therapy for up to 8 years of treatment.

## Limitations and strengths

Due to the partly retrospective design, many questionnaires and scales included in the protocol were not available at baseline especially in patients already in treatment with LCIG for more than 1 year. Therefore, the questionnaires with baseline assessment were available only in the smaller sample represented by the prospective population.

Since this was an observational study with the use of LCIG in routine care, the outcomes may be considered to be close to the real-world clinical practice, even if in the absence of a true control group.

Further limitation of this study is the fact that the results are not corrected for the levodopa equivalent daily dose of concomitant oral/transdermal antiparkinsonian medications. Although the use of concomitant medications was reduced after LCIG start, patients were still taking some antiparkinsonian or antidyskinetic during the study; therefore, improvements in dyskinesias and fluctuations may be also due an adjunctive effect.

The sub-analyses took into account the age of patients at the enrollment and not the age at LCIG implant, considering that age at treatment initiation is another important aspect in LCIG selection criteria. This would have allowed a comparison with the results obtained in an interesting prospective, open-label study in 28 patients with APD treated with LCIG for a mean treatment period of 24 months. In that study, younger age at operation and absence or presence of mild psychiatric/behavioral symptoms were positive predictive factors in selecting the best candidates for LCIG therapy [23].

## Conclusion

In conclusion, these results confirm that treatment with LCIG in the long term produces clinically significant improvements on motor function, non-motor symptoms, including sleep and impulsive disorders, and ADL in PD patients not optimally controlled by oral/transdermal therapies. This study also suggested a greater improvement in motor fluctuation and in ADL in advanced PD patients treated earlier since PD onset. Adverse effects and complications have been observed during the study period; therefore, the proper indications for LCIG should be always taken into account for the selection of a patient, considering the benefit against safety issues.

## Electronic supplementary material

Below is the link to the electronic supplementary material.

Supplementary file1 (DOCX 15 kb)
