# Supplementary material for: Motor and non-motor outcomes in patients with advanced Parkinson’s disease treated with levodopa/carbidopa intestinal gel: final results of the GREENFIELD observational study
Source: J Neurol. 2019 May 27;266(9):2164–76. doi: 10.1007/s00415-019-09337-6 (PMC6687881; doi:10.1007/s00415-019-09337-6)
Supplement: Supplementary file 1 — Supplementary file1 (DOCX 15 kb) [file 415_2019_9337_MOESM1_ESM.docx]

Supplementary Table 1: **Outcome of non-motor symptoms and PD associated symptoms at baseline and after LCIG at each treatment visit in retrospective patients**

ADL, activities of daily living; BL, baseline; LCIG; levodopa/carbidopa intestinal gel; SD, standard deviation; UPDRS, United Parkinson’s Disease Rating Scale.

| **RETROSPECTIVE WITHOUT LOCF** | **Baseline/V1**  **Mean ± SD** | **Visit 2**  **Mean ± SD** | **Visit 3**  **Mean ± SD** | **P ANOVA for repeated measures** |
| --- | --- | --- | --- | --- |
| **PDQ-39 (score 0-156)** | 69.7 ± 25.2 | 74.9 ± 25.3 | 76.7 ± 25.3 | P=0.008 |
| **PDSS-2 (score 0-60)** | 20.4 ± 10.3 | 21.2 ± 9.3 | 22.6 ± 10.2 | P=0.682 |
| **GFQ (score 0-64)** | 27.4 ± 12.0 | 27.8 ± 12.4 | 29.9 ± 13 | P=0.074 |
| **QUIP-RS (score 0-112)** | 8.8 ± 12.7 | 7.7 ± 11 | 8.7 ± 13.4 | 0.496 |
| QUIP-Gambling (score 0-16) | 1.3 ± 3.4 | 0.8 ± 2.6 | 1.5 ±3.9 | 0.779 |
| QUIP-Sexual behaviour | 1.1±2.6 | 1.2±2.8 | 1±2.5 | 0.367 |
| QUIP-Buying | 0.7±1.9 | 0.8±2 | 0.9±2.1 | 0.358 |
| QUIP-Eating | 2.5±4.1 | 2.4±4.3 | 2.6±4.4 | 0.838 |
| QUIP-Hobbism | 1.5±2.7 | 1.1±2.5 | 1±1.9 | 0.388 |
| QUIP-Punding | 0.9±2.1 | 0.9±1.9 | 0.8±2.4 | 0.345 |
| QUIP-Medication use | 0.8±2.4 | 0.6±1.8 | 0.9±2.8 | 0.862 |
| **RSS-2 (score 15-75)** | 38.8 ± 11.7 | 41.1 ± 11.5 | 40 ± 11.5 | 0.022 |
| RSS/Personal distress | 16.2 ± 4.7 | 17 ± 4.7 | 16.7 ± 4.6 | 0.190 |
| RSS/Negative feeling | 8.4 ± 3 | 9 ± 3 | 8.8 ± 3.2 | 0.030 |
| RSS/Life upset | 14.1 ± 5.6 | 15.1 ± 5.4 | 14.5 ± 5.4 | 0.039 |
